# Computer-Aided Drug Design and Drug Discovery: A Prospective Analysis

**DOI:** 10.3390/ph17010022

**Published:** 2023-12-22

**Authors:** Sarfaraz K. Niazi, Zamara Mariam

**Affiliations:** 1 College of Pharmacy, University of Illinois, Chicago, IL 60012, USA; 2 Centre for Health and Life Sciences, Coventry University, Coventry City CV1 5FB, UK

**Keywords:** Computer-Aided Drug Design (CADD), Machine Learning and Artificial Intelligence (AI), drug discovery, Chemoinformatics, molecular modeling, molecular docking, target identification

## Abstract

In the dynamic landscape of drug discovery, Computer-Aided Drug Design (CADD) emerges as a transformative force, bridging the realms of biology and technology. This paper overviews CADDs historical evolution, categorization into structure-based and ligand-based approaches, and its crucial role in rationalizing and expediting drug discovery. As CADD advances, incorporating diverse biological data and ensuring data privacy become paramount. Challenges persist, demanding the optimization of algorithms and robust ethical frameworks. Integrating Machine Learning and Artificial Intelligence amplifies CADDs predictive capabilities, yet ethical considerations and scalability challenges linger. Collaborative efforts and global initiatives, exemplified by platforms like Open-Source Malaria, underscore the democratization of drug discovery. The convergence of CADD with personalized medicine offers tailored therapeutic solutions, though ethical dilemmas and accessibility concerns must be navigated. Emerging technologies like quantum computing, immersive technologies, and green chemistry promise to redefine the future of CADD. The trajectory of CADD, marked by rapid advancements, anticipates challenges in ensuring accuracy, addressing biases in AI, and incorporating sustainability metrics. This paper concludes by highlighting the need for proactive measures in navigating the ethical, technological, and educational frontiers of CADD to shape a healthier, brighter future in drug discovery.

## 1. Introduction to Computer-Aided Drug Design (CADD)

### Computer-Aided Drug Design (CADD): A Synthesis of Biology and Technology

Identifying and developing a novel therapeutic agent can be an exhaustive and expensive endeavor in the expansive realm of drug discovery, where biology converges with chemistry. Historically, this journey relied on serendipitous discoveries or traditional trial-and-error methodologies, often consuming decades and substantial resources without a guaranteed outcome. The late 20th century heralded a transformative epoch for this field with the introduction of Computer-Aided Drug Design (CADD), which blends the intricate complexities of biological systems with the predictive power of computational algorithms and the development of chemical as well as biological-data-curated databases [1]. The core principle underpinning CADD are the utilization of computer algorithms on chemical and biological data to simulate and predict how a drug molecule will interact with its target—usually a protein or DNA sequence in the biological system [2]. This can range from understanding the drug’s molecular structure or target and predicting how the drug will bind to forecasting the pharmacological effects and potential side effects.

**Figure 1:**
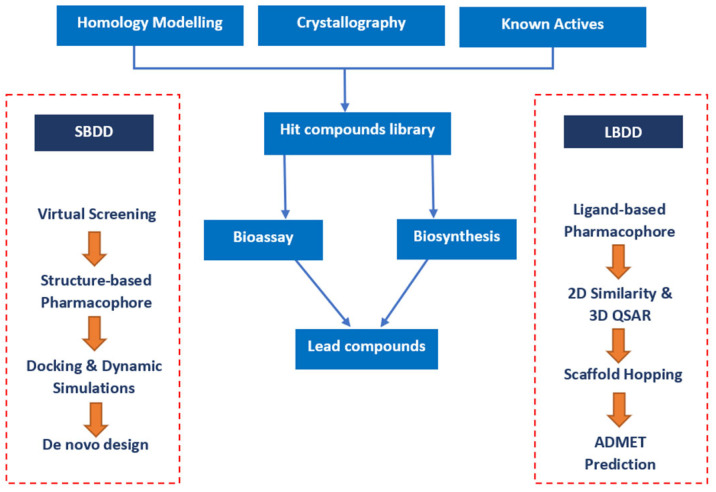
Conventional pathways in structure-based drug design (SBDD) and ligand-based drug design (LBDD) employ distinct methodologies. SBDD centers on target biomolecule structures, while LBDD relies on known ligand characteristics [7].

CADDs birth was facilitated by two crucial advancements: the blossoming field of structural biology, which unveiled the three-dimensional architectures of biomolecules, and the exponential growth in computational power, which made it feasible to perform complex simulations in relatively shorter timeframes [3]. One of the earliest and most celebrated applications of CADD was in the design of the anti-influenza drug Zanamivir. This process showcased the potential of this approach to significantly truncate the drug discovery timeline [4]. At its core, CADD is subdivided into two main categories: structure-based drug design (SBDD) and ligand-based drug design (LBDD) [5]. SBDD leverages knowledge of the three-dimensional structure of the biological target, aiming to understand how potential drugs can fit and interact with it. In contrast, LBDD does not require knowledge of the target structure but instead focuses on known drug molecules and their pharmacological profiles to design new drug candidates (Figure 1) [6].

The rise of CADD is synonymous with the paradigm shift in drug discovery, where the process transitioned from being largely empirical to becoming more rational and targeted [8]. However, as with any scientific methodology, CADD has challenges. While predicting the behavior of biological systems solely based on computer simulations, it is important to acknowledge the inherent pitfalls. For instance, consider the hypothetical scenario where a computer simulation accurately models the biochemical interactions between a receptor and its target. However, if the simulation lacks crucial real-world data on external environmental factors or unexpected biological responses, the predictions may deviate significantly from the actual outcomes. These models, while sophisticated, often require experimental validation to ascertain their predictions [9,10]. In conclusion, CADD signifies the harmonious blend of biology and technology, aiming to expedite drug discovery. While it has already made significant strides in the field, its full potential is yet to be realized as newer computational methods and an increased understanding of biological systems come to the fore.

## 2. Key Techniques and Approaches in CADD

### Delineating the Array of Techniques in Computer-Aided Drug Design

**Figure 2:**
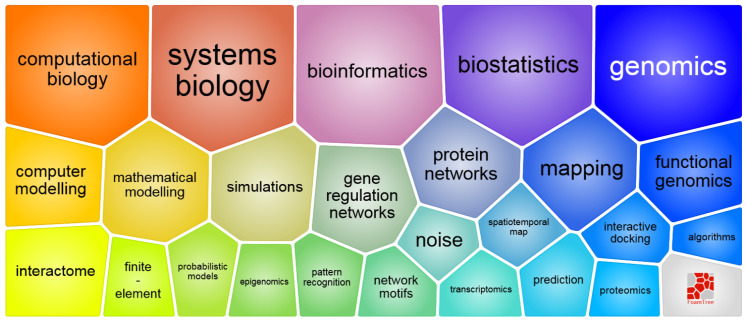
Elements of processing biomolecular data for CADD (image sourced from the European Research Council website [13]).

Computer-Aided Drug Design (CADD) is a powerful and interdisciplinary field that plays a pivotal role in modern drug discovery. It combines computational techniques with biological knowledge to identify and optimize potential drug candidates. This integration of diverse methodologies contributes to the versatility and effectiveness of CADD in the pharmaceutical industry. The vastness and versatility of CADD arise from the plethora of techniques and methodologies that underpin this field. This field’s effectiveness is rooted in its diverse methodologies, ranging from molecular modeling to predicting drug metabolism. Within CADD, adherence to Lipinski’s rule is paramount for achieving optimal oral drug likeliness, where compounds ideally minimize violations of criteria such as molecular weight, lipophilicity, hydrogen bond donors, and acceptors. This strategic integration of CADD principles and adherence to drug-likeness criteria collectively accelerates and refines the drug discovery process, showcasing the versatility and impactful role of CADD in various fields and the pharmaceutical industry (Figure 2) [11,12].

Molecular Modeling: At the heart of CADD lies molecular modeling, which encompasses a wide range of computational techniques used to model or mimic the behavior of molecules. This involves creating three-dimensional models of molecular structures, often of proteins and ligands. This technique provides insights into molecules’ structural and functional attributes, facilitating a deeper understanding of how potential drugs might behave within the biological system [14]. It enables researchers to visualize and analyze the interactions between drug candidates and their target proteins, aiding in the design and optimization of potential drugs. Recently developed AI/ML-driven tools like AlphaFold2 [15], trTosetta [16,17], Robetta [18], RoseTTA Fold [19], ESMFold [20], and OmegaFold [21] have accelerated protein structure prediction by many folds [22]. Methods like molecular dynamics (MD) simulations can forecast the time-dependent behavior of molecules, capturing their motions and interactions over time through various tools like Gromacs [23], ACEMD [24], and OpenMM [25,26] (Table 1).

Docking and Virtual Screening: Docking involves predicting the orientation and position of a drug molecule when it binds to its target protein. It estimates the binding affinity between the drug and its target, which is crucial in drug design [27]. Utilizing advanced tools such as AutoDock Vina [28], AutoDock GOLD [29], Glide [30], DOCK [31], LigandFit [32], and SwissDock [33], researchers can predict binding affinities and orientations with precision (Table 2). Conversely, virtual screening, a complementary approach, involves sifting through vast compound libraries to identify potential drug candidates [34]. Tools like DOCK [26], LigandFit [27], and ChemBioServer [35] facilitate this process, rapidly evaluating interactions and identifying compounds with high binding affinities. DOCK is renowned for structure-based drug design; LigandFit integrates into the Schrödinger suite; and ChemBioServer is an online platform for efficient virtual screening. The synergy of these docking and virtual screening tools enhances the accuracy of predictions, contributing significantly to the identification of promising drug candidates in the complex landscape of computational drug design. Many researchers, like Pinzi and Sohoo, have extensively discussed using these tools, showcasing their implementation in advancing the field of computational drug design [36,37].

Quantitative Structure-Activity Relationship (QSAR): QSAR modeling explores the relationship between the chemical structure of molecules and their biological activities. Through statistical methods, QSAR models can predict the pharmacological activity of new compounds based on their structural attributes, enabling chemists to make informed modifications to enhance a drug’s potency or reduce its side effects [38,39]. In a research endeavor by Luo et al., the Similarity Ensemble Approach (SEA) served as a pivotal tool employed to gauge the precision of k-nearest neighbors (kNN) Quantitative Structure-Activity Relationship (QSAR) models. These models were systematically constructed for known ligands associated with individual G Protein-Coupled Receptor (GPCR) targets to unveil active and inactive molecules [40]. Meanwhile, a separate investigation by Raj et al. focused on developing QSAR models for 50 compounds exhibiting anti-HIV activity utilizing the molecular field analysis method. The findings underscored the critical role of electrostatic and steric interactions in influencing the anti-HIV activity of the compounds [41]. Furthermore, a novel approach was adopted in another study leveraging a deep neural network by Nigsch et al., in conjunction with QSAR models, to analyze a diverse collection of 1000 chemicals known for their anti-cancer activity. According to this study, integrating the QSAR approach with deep learning techniques proved advantageous, enabling the identification of critical structural characteristics that significantly contributed to the compounds’ anti-cancer efficacy [42].

Pharmacophore Modeling: A pharmacophore is a spatial arrangement of essential features in a molecule necessary for its pharmacological activity. Pharmacophore modeling is a fundamental component of contemporary drug discovery, involving the identification of spatial arrangements of essential molecular features crucial for a molecule’s pharmacological activity. This approach is potent for medicinal chemists, enabling the rational design of novel compounds with optimized pharmacological properties [43].

For example, pharmacophore modeling has proven instrumental in kinase inhibitors. Zhang et al. utilized pharmacophore modeling to discern essential features in active kinase inhibitors, including hydrogen bond donors, acceptors, and hydrophobic regions. The identified pharmacophore elements provided valuable guidance for designing novel compounds, resulting in improved selectivity and potency against specific kinases implicated in disease pathways [44]. Similarly, pharmacophore modeling has been applied to design ligands targeting G protein-coupled receptors (GPCRs). Fidom et al. exemplified this application by elucidating the spatial arrangement of features crucial for GPCR binding, such as aromatic interactions and hydrogen bonding [45,46]. The resulting pharmacophore models facilitated the development of ligands with enhanced affinity and selectivity for specific GPCRs involved in diverse therapeutic areas. In summary, the strategic use of pharmacophore modeling enables systematic analysis of the essential features contributing to a molecule’s pharmacological activity. This knowledge enhances understanding of ligand-receptor interactions and empowers researchers to rationally design compounds with enhanced efficacy and reduced side effects, shaping the future landscape of pharmaceutical research.

Prediction of Drug Metabolism and Pharmacokinetics (DMPK): The ultimate success of a drug is not solely determined by its ability to bind to its target. Its metabolic stability, solubility, and how it is distributed in the body (pharmacokinetics) play pivotal roles. CADD offers tools that can predict the DMPK properties of compounds, allowing researchers to anticipate and address potential issues related to drug metabolism, bioavailability, and potential drug-drug interactions [47].

Novo Drug Design: Unlike other methods that modify existing molecules, de novo drug design creates new drug molecules from scratch. This technique leverages computational algorithms to generate new molecular structures that fit specific criteria, opening the door to many novel drug candidates [48].

In summary, the techniques embedded within CADD provide an integrated, multi-faceted approach to drug discovery. By offering a suite of tools that spans molecular modeling to drug metabolism prediction, CADD ensures that drug candidates are potent and selective and have optimal pharmacokinetic and safety profiles.

## 3. Integration of Machine Learning and AI in CADD

### 3.1. Machine Learning and AI: The New Vanguard in Drug Discovery

**Figure 3:**
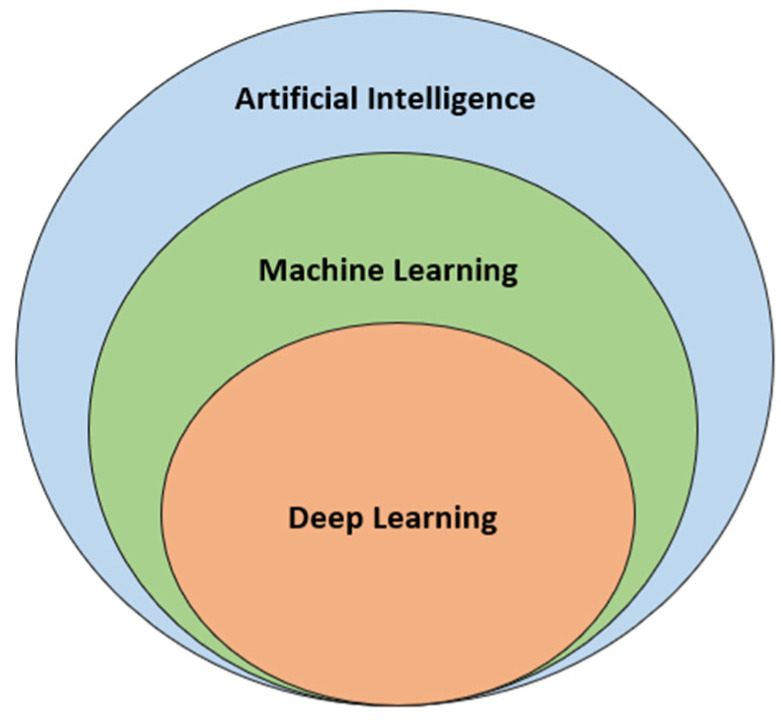
Subsets of Artificial Intelligence: Machine Learning and Deep Learning.

The technological renaissance that defines the 21st century has borne witness to the meteoric rise of Machine Learning (ML) and Artificial Intelligence (AI). These computational realms, known for their data-driven decision-making capabilities, have begun to significantly influence the sphere of Computer-Aided Drug Design (CADD), reshaping the contours of drug discovery [49]. Machine Learning, a subset of AI, hinges on algorithms that can learn patterns from vast data sets without being explicitly programmed for specific tasks (Figure 3) [50]. In drug discovery, ML has been instrumental in predicting molecular properties, understanding drug-receptor interactions, and forecasting biological responses based on chemical structures. Techniques such as deep learning, which uses neural networks modeled after the human brain, show immense potential for predicting complex drug-related outcomes with remarkable accuracy [51].

### 3.2. Implications of ML in CADD

Predicting Drug-Drug Interactions: One of the challenges in drug discovery is understanding how a new drug might interact with other medications a patient might be taking. ML algorithms can process large databases of known drug-drug interactions to predict potential harmful combinations for novel compounds [52].

Drug Repurposing: Drug repurposing involves finding new therapeutic applications for existing drugs. By analyzing vast datasets, Machine Learning can identify potential new targets for existing medications, thus saving both time and costs associated with traditional drug discovery [53].

Generative Adversarial Networks (GANs) in Drug Design: GANs are a form of AI where two neural networks (a generator and a discriminator) are trained in tandem. The generator creates molecular structures while the discriminator evaluates them. Over time, the generator becomes adept at creating feasible and potentially bioactive molecular structures, which can be synthesized and tested in the lab [54].

Predictive Toxicology: One of the primary reasons drug candidates fail in clinical trials is unforeseen toxicity. ML models can help predict potential adverse effects by analyzing historical data on drug-induced toxicities, thus filtering out potentially toxic compounds early in the discovery process. Furthermore, utilizing descriptors like molecular weight, lipophilicity, and electronic properties, QSAR models predict toxicological effects by correlating a molecule’s structure with its potential toxicity. Additional descriptors, such as solubility, metabolic stability, and identification of toxicophores, provide comprehensive insights, facilitating early hazard identification and prioritization of compounds for experimental testing in computational toxicology.

The integration of AI and ML into CADD signifies more than just the adoption of new technologies. It represents a paradigm shift from traditional hypothesis-driven research to data-driven discovery, leveraging the power of big data and computational prowess to inform decision-making at every step of drug discovery [55]. However, while these technologies promise a revolution in drug discovery, challenges persist. Issues such as data quality, interpretability of AI models, and the need for experimental validation continue to be focal areas of attention in this integration [56]. In essence, the synergy of ML, AI, and CADD sets the stage for a new era in drug discovery. An era characterized by increased efficiency, reduced costs, and the rapid delivery of effective therapeutics to patients in need.

## 4. Challenges and Limitations in CADD

### Understanding the Obstacles: The Roadblocks in Computer-Aided Drug Design

While CADD offers unparalleled advantages in expediting and refining drug discovery, it is crucial to recognize its inherent challenges. A notable obstacle is the scarcity of experts proficient in AI/ML within CADD. Initiatives like specialized training programs and targeted recruitment are crucial; for example, organizations like in-silico Medicine are pioneering efforts to bridge this gap, fostering a skilled workforce capable of harnessing advanced computational techniques for drug discovery. Addressing these limitations can lead to better strategies and pave the way for more effective drug discovery workflows [57].

Accuracy of Predictive Models: In CADD, a major challenge lies in ensuring the accuracy of computational models, given that molecular dynamics simulations, docking scores, and machine learning predictions all rely on theoretical models. These models may not fully capture the intricate nuances of biological systems. To enhance accuracy, it is essential to delve into the intricacies of scoring algorithms [58]. Scoring algorithms in drug discovery are pivotal for predicting the binding affinity between molecules and their targets. To ensure their accuracy, it is imperative to actively mitigate the risk of false positives and negatives. This involves meticulous calibration of scoring parameters, the incorporation of diverse molecular descriptors, and continuous validation against experimental data. For instance, refining docking scores through rigorous validation against known binding affinities can enhance the reliability of predictions. By optimizing the balance between sensitivity and specificity, researchers can bolster confidence in scoring algorithms, reducing the likelihood of inaccuracies in drug discovery predictions [59,60,61].

Data Quality and Quantity: The predictions made by CADD tools are only as good as the data they are trained on. The predictions are likely inaccurate if the underlying data are of poor quality or insufficient. The lack of curated, high-quality datasets, especially in the context of machine learning in drug discovery, is a recurring challenge [62]. Removing outliers and ensuring consistent data formatting can refine molecular interaction datasets, minimizing inaccuracies and bolstering the reliability of computational models. Additionally, implementing standardized experimental protocols, such as consistent assay conditions and endpoint measurements, further contributes to improved data quality in CADD, ensuring robust and dependable results.

Over-reliance on Computational Predictions: While CADD is a powerful tool, over-reliance on its predictions without subsequent experimental validation can lead to misguided efforts. Balancing computational predictions with experimental evidence is essential for a successful drug discovery [63].

Time and Computational Cost: Some advanced CADD techniques, especially those involving extensive molecular dynamics simulations or intricate machine learning models, require vast computational resources. The associated costs, both in terms of time and infrastructure, can be prohibitive for some research groups [64].

Representing Molecular Flexibility: Most biological molecules, including potential drug compounds and their target proteins, are highly flexible. Accurately representing this flexibility, especially in techniques like molecular docking, is challenging and can significantly impact the results of CADD studies [65].

Interpretability of AI Models: As AI and machine learning models become more complex, their predictions become more challenging to interpret. This ‘black-box’ nature of AI models can make it challenging to understand why a particular compound is predicted to be active or how its structure might be optimized [66].

Despite these challenges, the potential benefits of CADD in drug discovery are immense. By acknowledging these limitations and continually striving to address them through innovation and research, CADD will remain at the forefront of modern drug discovery, shaping the future of therapeutics.

## 5. Experimental Validation in CADD: From In-Silico to the Lab Bench

### Bridging Computational Predictions with Reality

At the crossroads of drug discovery, Computer-Aided Drug Design (CADD) outputs demand rigorous experimental validation to ensure their biological and therapeutic relevance. A drug’s true potential can only be ascertained through this synergy between the computational and experimental realms [67].

No matter how advanced, computational predictions are inherently rooted in theoretical models. While these models can approximate biological systems, discrepancies always exist. Experimental validation serves as the crucible, determining whether a predicted molecule has genuine therapeutic promise or is merely a computational artifact [68]. After the CADD process identifies potential drug candidates, biochemical assays often serve as the first validation step. Such assays measure the interaction between the proposed drug molecule and its intended target protein, offering insights into binding affinities and possible mechanisms of action [69]. Cell-based assays are employed to further understand a drug’s biological relevance. These tests assess how a compound affects cellular functions, allowing researchers to ascertain its potential efficacy and toxicity in a more complex, biologically relevant setting [70]. Before any drug candidate reaches human trials, its efficacy, safety, and pharmacokinetic properties must be investigated in vivo. Animal models serve this purpose, providing a more comprehensive understanding of how a drug will behave in a living organism [71].

Techniques such as X-ray crystallography and NMR spectroscopy can provide atomic-level details of the interaction between a drug and its target. Such insights can validate computational predictions, refine drug design strategies, and offer mechanistic understandings of drug action [72]. Often, experimental validation reveals unexpected outcomes or unanticipated challenges. Rather than being a linear process, drug discovery often involves iterations between CADD predictions and experimental testing, leading to refined models and better drug candidates [73].

Furthermore, to ensure robust and validated results in CADD, the initial imperative is to employ software acknowledged for its accuracy in scoring results in simulations and predictions. Utilizing well-validated tools, such as AutoDock Vina or Schrödinger Suite, establishes a foundation of reliability and precision in the computational models, laying the groundwork for more dependable and meaningful insights in CADD simulations and predictions.

In essence, while CADD provides a powerful arsenal of tools to guide and expedite drug discovery, the proof of a drug’s worth always rests in the experimental realm. This synergy between computation and experimentation forms the backbone of modern drug discovery, ensuring that only the most promising compounds transition from the digital domain to the bedside.

## 6. Harnessing the Power of AI: A Paradigm Shift in Drug Discovery

The infusion of artificial intelligence (AI) and machine learning (ML) into the realm of Computer-Aided Drug Design (CADD) represents one of the most significant shifts in modern drug discovery methodologies. These computational methods promise unprecedented speed, accuracy, and insights into the complex dance of molecular interactions [74]. Machine learning, a subset of AI, has experienced a surge in its application to drug discovery. Unlike traditional CADD methods that rely on predefined algorithms to predict molecular behavior, ML algorithms learn from data, enhancing their predictive power with each iteration [75].

One of MLs strengths in CADD is its ability to extract patterns and knowledge from vast datasets. With the exponential growth of biomedical data, ML models, especially deep learning architectures, can identify complex relationships and features that might be non-intuitive to researchers [76]. The ML models have been instrumental in predicting drug responses based on molecular structures, pharmacological profiles, and even genetic data. Additionally, they offer insights into potential drug-drug interactions, a critical aspect of ensuring drug safety [77]. Recently, advanced ML models, such as Generative Adversarial Networks (GANs) and Variational Autoencoders (VAEs), have been employed to generate novel molecular structures that could be potential drug candidates, merging the worlds of creativity and computation [78]. 

Machine learning models, especially deep learning, can manage high-dimensional data and circumvent some traditional CADD limitations, such as the need for extensive feature engineering [79]. While AI and ML have immense potential, they also raise concerns. The “black box” nature of some deep learning models challenges interpretability, which is crucial for scientific rigor. Ethical considerations arise significantly when leveraging patient data for model training [80]. In conclusion, as CADD embraces the AI revolution, the drug discovery landscape is poised for transformative changes. Ensuring the responsible and effective integration of these technologies will dictate the trajectory of future therapeutic breakthroughs.

## 7. Integration of Multi-Omics Data in CADD

### Holistic Viewpoints: Embracing the Complexity of Biology through Multi-Omic Integration

The biological systems underlying disease states and drug interactions are intricate, with layers of regulation and interplay. A comprehensive understanding necessitates analyzing not just one but multiple “omes”—the genome, transcriptome, proteome, and metabolome, among others. 

Integrating this multi-omics data into CADD ensures a more holistic approach to drug discovery [81]. Single-omics studies, while informative, offer just a glimpse of the biological puzzle. By combining multiple layers of omics data, researchers can gain a more comprehensive view of disease states, potential drug targets, and overall cellular dynamics [82]. Genomic data offers insights into the likely genetic drivers of diseases. When integrated into CADD, this information can guide the search for drug targets, especially in conditions with a strong genetic component, like certain cancers [83].

The transcriptome represents all RNA molecules in a cell, reflecting genes actively being transcribed. Integrating transcriptomic data can offer insights into how cells might respond to a drug at the mRNA level, even hinting at potential side effects or alternate pathways [84]. While genes and transcripts are crucial, proteins are often the direct targets of drugs. Proteomic data can help understand drug-protein interactions, post-translational modifications, and potential off-target effects [85].

Metabolomics, the study of small molecules in biological systems, offers vital information on a drug’s metabolism, its interactions with endogenous metabolites, and potential biomarkers for drug efficacy and toxicity [86]. Beyond examining individual omics layers, systems biology takes a more integrative approach. By constructing networks of interactions based on multi-omics data, researchers can predict how drugs might affect entire pathways or networks, leading to a more systemic understanding of drug action [87]. While each “omics” layer provides invaluable insights, their combination can revolutionize CADD. By embracing the complexity of biology through multi-omics integration, drug discovery can move closer to more effective and personalized therapeutic solutions.

## 8. Current Challenges in CADD

### Overcoming Barriers: The Evolving Landscape of Challenges in Computer-Aided Drug Design

While the advancements in Computer-Aided Drug Design (CADD) have revolutionized drug discovery, the field is not without its challenges. From data quality to the need for more predictive models, these hurdles highlight areas ripe for further innovation [63]. One of the most fundamental challenges is the quality and availability of data. Inaccuracies in datasets, such as incorrect compound structures or misleading bioactivity data, can misguide computational predictions. Furthermore, proprietary data hoarding limits the sharing and consolidation of knowledge [88]. Despite progress, there is a continual need for models with better predictive power. Particularly in drug-target interaction predictions, models can sometimes produce false positives or overlook viable candidates [89].

Proteins, nucleic acids, and other biological macromolecules are dynamic. Accounting for this flexibility in simulations, especially over long timescales, remains a significant computational challenge [90]. As drug databases grow and models become more intricate, ensuring that CADD methods scale effectively is crucial. This requires continual optimization of algorithms and leveraging advanced computational infrastructure [91]. With the influx of multi-omics and diverse biological data, integrating these heterogeneous datasets in a meaningful manner that enhances drug discovery is a non-trivial task [92]. 

As CADD often leverages patient data, especially in personalized medicine, ensuring data privacy and addressing ethical concerns associated with data usage are paramount [93]. In summary, while CADD continues to propel drug discovery into the future, addressing its challenges is essential. The field can evolve, adapt, and continue its trajectory toward more efficient and effective drug discovery paradigms by confronting these obstacles head-on.

## 9. Case Studies: Success Stories in CADD

### From Concept to Clinic: Triumphs in Computer-Aided Drug Design

The real impact of any scientific discipline can often be best appreciated through tangible success stories. In Computer-Aided Drug Design (CADD), several compounds have transitioned from the computer screen to clinical applications, underscoring the potential of computational approaches [94].

HIV Protease Inhibitors: The battle against HIV/AIDS saw a significant leap with the development of protease inhibitors. CADD played a pivotal role, especially in the development of drugs like saquinavir. Through molecular modeling and simulation, researchers identified potential binding pockets, paving the way for more targeted drug development [95].

Anti-influenza Drugs: The neuraminidase inhibitors, specifically oseltamivir (Tamiflu), were developed using structure-based drug design. By analyzing the protein structures of influenza strains, computational models aided in pinpointing drug targets, eventually leading to effective flu treatments [96].

Imatinib and Chronic Myeloid Leukemia: A revolutionary drug in treating Chronic Myeloid Leukemia, imatinib’s (Gleevec) development was bolstered by CADD. By targeting the BCR-ABL kinase, imatinib exemplifies how computational insights can lead to potent and selective inhibitors [97].

HCV Protease Inhibitors: Hepatitis C was once a challenging disease to treat. The introduction of drugs like boceprevir, developed with significant CADD input, transformed HCV therapy. Through molecular dynamics and docking studies, researchers have identified inhibitors targeting HCV protease [98].

Alzheimer’s Disease and β-secretase Inhibitors: While the battle against Alzheimer’s is ongoing, CADD has contributed to the development of potential treatments. By targeting the β-secretase enzyme, inhibitors have been computationally designed, some of which have progressed to clinical trials, i.e., Donanemab and Solanezumab [99,100,101].

SARS-CoV: In response to the COVID-19 pandemic, researchers leveraged advanced CADD methods for lead identification for potential antiviral drugs. Investigations into flavonoid glycosides in medicinal plants revealed their potential protective effects against COVID-19 infections. Docking studies with alkaloids and vanillin derivatives suggested possible inhibition of SARS-CoV-2. Furthermore, modeling studies encompassing ligand-based and structure-based activities provided valuable insights, highlighting the multidimensional approach of computational chemistry in the quest for effective treatments against COVID-19 [102].

In reflection, these success stories embody the essence of CADDs potential in modern drug discovery. They represent hope, progress, and a testament to the synergy of computational methods and medicinal chemistry.

## 10. The Future of CADD: Emerging Technologies and Innovations

### 10.1. Charting the Horizon: Navigating the Next Frontiers of Computer-Aided Drug Design

The transformative influence of CADD on drug discovery is beyond dispute. However, like any evolving discipline, the future holds new challenges and unparalleled opportunities. Harnessing cutting-edge technologies and paradigms can unlock an era where drug discovery is faster, more precise, and more patient-centric [103]. Traditional computing faces limitations in handling complex drug design problems. Quantum computing, with its ability to control and compute information radically differently, may revolutionize molecular modeling and simulations, enabling the exploration of vast molecular spaces in mere seconds [104]. Immersive technologies can provide researchers with an intuitive understanding of molecular structures and interactions. Through AR/VR, drug design can become a more tactile and visual endeavor, enhancing molecular modeling and collaborative efforts [105]. Machine learning, notably deep learning, is rapidly becoming integral to CADD. Neural networks, with their ability to recognize patterns from vast datasets, can predict drug interaction toxicity and suggest novel drug compounds [106].

As genomic sequencing becomes more commonplace, CADD tools that cater to individual genetic profiles will gain prominence. This will foster an era of genuinely personalized drugs tailored to an individual’s genetic makeup [107]. Open-source and collaborative platforms can democratize drug discovery. By harnessing the collective intelligence of the global scientific community, these platforms can accelerate the drug discovery process and integrate diverse expertise [108].

As environmental concerns come to the fore, integrating principles of green chemistry into CADD can result in drug synthesis processes that are both efficient and environmentally benign [109]. In the grand vista of drug discovery, the future of CADD shines bright. Embracing innovations and pushing the boundaries of technology will enhance the discipline and promise a better healthcare future for all.

### 10.2. Unity in Diversity: Harnessing Global Intelligence in Computer-Aided Drug Design

In a progressively interconnected world, the role of collaborative networks and open-source platforms in CADD cannot be overstated. These entities amplify the collective intellectual prowess of researchers worldwide, allowing for a swift, democratic, and cost-efficient drug discovery process [110]. Traditional drug discovery often demands vast resources, making it an exclusive venture. Open-source platforms democratize this, allowing researchers to contribute and access advanced CADD tools [111] irrespective of their affiliations. Initiatives like the Open-Source Drug Discovery (OSDD) project for tuberculosis exemplify this global commitment [112].

Crowdsourcing platforms in CADD harness the power of global intellect. Challenges posted on these platforms lead to diverse solution pathways, many of which might be non-traditional yet highly effective [113]. Open-source platforms ensure that CADD tools are continually improved. Community-driven tools are updated frequently based on user feedback and the latest scientific advancements [114,115,116]. In an age characterized by collaboration and open access, collaborative networks and open-source platforms in CADD emerge as beacons of hope. They underline the belief that in unity lies strength, and in shared knowledge lies the promise of a healthier tomorrow.

### 10.3. Drawing Lines in the Digital Sand: Navigating the Ethical and Regulatory Labyrinths of Computer-Aided Drug Design

In the exhilarating race of drug discovery through CADD, the underlying ethical and regulatory considerations provide crucial checkpoints. Ensuring these digital methodologies hasten drug discovery and preserving the highest ethical standards becomes paramount [117].

With the increased utilization of patient data in personalized medicine, ensuring data privacy are paramount. Regulations like the General Data Protection Regulation (GDPR) guide the collecting, storing, and processing of personal data in research, imposing stringent data protection requirements [118]. Defining IP rights can become murky as CADD veers towards more collaborative and open-source models. Balancing between open-access and proprietary claims ensures researchers and institutions obtained due credit [119]. AI-driven methodologies in CADD can sometimes inherit biases present in their training data. Ensuring that these models are transparent, interpretable, and unbiased becomes essential for ethical drug discovery [120]. Reproducibility, a cornerstone of scientific rigor, must be confirmed in CADD. Ensuring consistent results across different computational settings is pivotal [121] with increasingly complex algorithms and models.

While CADD can predict potential drug candidates, the transition to in vivo testing, especially on animals, brings its own set of ethical concerns. Regulatory bodies provide guidelines on minimizing animal testing and ensuring humane conditions [122]. For a drug to reach the market, it is not enough for it to be discovered through CADD; regulatory bodies must accept and validate these methodologies. Collaborations between CADD scientists and regulatory authorities can streamline this acceptance process [123]. In conclusion, while CADD offers transformative potential in drug discovery, it is essential to navigate the process with ethical integrity and in compliance with existing regulations. As the adage goes, with great power comes great responsibility, and in the realm of CADD, this holds especially true.

### 10.4. A Glimpse into the Horizon: Envisioning the Next Epoch of Computer-Aided Drug Design

The ever-evolving realm of CADD continues to offer promise and innovation. However, as with any cutting-edge field, it is fraught with challenges and uncertainties. Looking forward, it is essential to pinpoint potential trajectories and hurdles that might shape the next generation of drug discovery [124]. As we stand on the brink of a quantum revolution, the potential for quantum computers to optimize molecular simulations and improve drug design methodologies is immense. They promise speed and precision previously deemed unattainable [104]. The continued evolution of AI promises more sophisticated drug discovery models. Deep learning models that can simulate protein folding or predict drug-target interactions with increased accuracy are on the horizon [125]. With advancements in genomics, proteomics, and metabolomics, integrating this vast and varied data into CADD will allow for a more holistic approach to drug design, considering intricate biological systems [81]. As the volume of biomedical data explodes, standardizing this data to ensure consistency and reliability in CADD methodologies becomes a significant challenge [126]. The ecological footprint of drug development cannot be ignored. Future CADD models might need to incorporate sustainability metrics, ensuring that drug discovery does not come at an environmental cost [127]. As AI becomes more prominent in drug discovery, ethical concerns about machine autonomy, transparency in algorithmic decisions, and potential biases become more pronounced [128]. In essence, the future of CADD is an intricate tapestry of innovation, challenges, and ethical considerations. By preemptively addressing these challenges and harnessing new technologies, CADD can continue revolutionizing drug discovery, ensuring better health outcomes for all.

## 11. Bridging the Gap: Integrating Experimental Data with CADD

### 11.1. Forging Synergy: When the Computational Meets the Experimental in Drug Design

As the chasm between experimental biology and computational methodologies in drug design narrows, the symbiosis between these disciplines offers unparalleled potential. While CADD provides the tools to forecast and simulate, experimental data acts as both the foundation and the validator of these predictions [129]. While CADD can predict a myriad of drug properties, these remain theoretical until experimentally verified. Experimental results offer evidence of drug efficacy, metabolism, and safety, among other characteristics [130]. Experimental data does not just validate CADD predictions; it also enriches them. This data are invaluable when a predicted molecule does not yield the expected results in the lab. It informs subsequent design iterations, leading to a more refined and likely successful candidate [131].

Molecular dynamics simulations can predict how molecules will behave over time. Yet, experimental techniques like X-ray crystallography or nuclear magnetic resonance (NMR) provide snapshots of these molecules, which can validate or recalibrate these simulations [132]. Experimental results from high-throughput screenings, assays, and other methodologies provide a wealth of data. This data can be mined using AI and other CADD tools to uncover patterns, relationships, or potential drug candidates that might be overlooked [133]. With a growing database of experimental results, the predictive models used in CADD can be trained more effectively. This integration helps continually refine the accuracy of CADD models, making them more reliable over time [134].

While CADD offers tools to navigate the complex maze of biological systems, real-world experimental data provides the actual map. Together, they offer a more straightforward path to successful drug candidates [135]. In sum, the confluence of experimental data and CADD are more than just complementary; it is synergistic. Drug discovery becomes more robust, efficient, and accurate by fostering a more intimate relationship between these domains.

### 11.2. Shaping the Drug Designers of Tomorrow: The Essentiality of CADD in Modern Education

The realm of drug discovery, rife with promise, demands cutting-edge technology and well-equipped minds to wield it. As CADD emerges as a linchpin in the drug discovery landscape, it underscores the urgency of integrating CADD training into contemporary education [136].

While traditional chemistry and biology programs emphasize foundational knowledge, introducing CADD modules can offer students early exposure to the computational aspects of drug design. Such foundational exposure can spark interest and cultivate the next generation of drug discoverers [137]. Universities worldwide are realizing the importance of specialized courses focusing solely on CADD. These courses amalgamate computational methodologies, biology, and drug pharmacology, producing experts capable of spearheading drug discovery ventures [138]. The volatile, evolving nature of CADD mandates professionals to be in a perpetual state of learning. Workshops, online courses, and conferences focusing on the latest CADD methodologies are indispensable for professionals to stay abreast of [139].

Drug design is a symphony of various disciplines. Ensuring that CADD training is not siloed but integrates elements of biology, chemistry, AI, and even ethics is crucial. A holistic, multidisciplinary approach produces well-rounded professionals [140]. Encouragingly, many institutions offer research opportunities focused on CADD for postgraduates and early-career scientists. These platforms allow hands-on experience, bridging the gap between theory and real-world applications [141]. The pharmaceutical and biotech industries have a vested interest in the proficiency of CADD professionals. Collaboration between academia and industry can drive curriculum development, ensuring it aligns with the real-world demands of drug discovery [142]. Conclusively, as the tower of drug discovery leans more on CADD, training proficient individuals becomes paramount. An investment in education is an investment in a healthier, brighter future.

## 12. The Future Outlook: CADDs Trajectory and Upcoming Challenges

The rapid progression of CADD, coupled with its integral role in recent drug discoveries, prompts us to ponder the trajectory of this discipline and the challenges it is poised to encounter [143]. With quantum computers inching closer to practical applications, their potential impact on CADD is enormous. Quantum algorithms can drastically reduce the time required for molecular simulations, thereby accelerating drug discovery manifolds [104].

While AI and machine learning have already entrenched themselves in CADD, the proliferation of deep learning models promises even more precise predictions. These models, trained on vast datasets, might eventually surpass traditional simulation methods in accuracy [144]. With advances in biology, previously deemed ‘undruggable’ targets are now within CADDs crosshairs. This shift demands that CADD evolve and devise strategies to engage with these challenging targets [145]. As CADD and AI models start playing more prominent roles in determining drug viability, ethical questions about trustworthiness, bias in predictions, and accountability will arise. Addressing these concerns will be paramount [146]. With genomics, proteomics, and metabolomics offering a deluge of biological data, CADDs future lies in efficiently harnessing this data. Integrating multi-omics data can provide a holistic view of biological systems, facilitating better drug design [147].

Furthermore, in addition to extensive datasets, the execution of numerous simulation processes in AI/ML necessitates high-specification hardware. The computational demands of AI/ML algorithms, such as deep learning models, require robust hardware configurations with powerful processors, ample memory, and efficient GPUs to handle complex computations effectively. Access to high-performance hardware is crucial for optimizing the training and inference phases of AI/ML systems, ensuring the timely and accurate processing of tasks.

As collaborative efforts become more common, ensuring the privacy and security of shared data becomes critical. Developing protocols and standards for data sharing without compromising data security will be pivotal [148]. The environmental footprint of drug discovery, especially with energy-intensive computational methods, cannot be ignored. Future CADD methodologies must be sustainable, considering drug efficacy and environmental impact [149]. In essence, while the future of CADD radiates promise, it is not without its challenges. Navigating this labyrinth will necessitate a fusion of technological prowess, ethical considerations, and a commitment to sustainable practices.

## 13. Collaborative Efforts and Global Initiatives in CADD

### Bridging Boundaries: How Global Collaborations Are Amplifying the Impact of CADD

The challenges associated with drug discovery are monumental, often transcending the capacities of individual institutions or nations. Recognizing this, a wave of collaborative efforts and global initiatives in CADD have been established, pooling resources, expertise, and data for a common goal [150]. Platforms such as Open-Source Malaria and OpenZika are pioneering the democratization of drug discovery. These platforms catalyze widespread participation and foster innovation by making research data and tools available to the public [151]. Collaborative groups, such as the Innovative Medicines Initiative (IMI) and Structural Genomics Consortium, bring together academia, industry, and nonprofits. Such consortia streamline research efforts, prevent redundancy, and accelerate discovery [152]. The significance of sharing molecular databases, software tools, and algorithms cannot be overstated. Initiatives like PubChem, ChemSpider, and the Protein Data Bank serve as repositories that are invaluable for researchers across the globe [153].

Cloud platforms like IBMs Watson for Drug Discovery allow shared computational resources, enabling small research groups to undertake large-scale simulations without colossal infrastructure investments [154]. Leading universities often engage in collaborative research programs, benefiting from shared expertise, resources, and diversified perspectives. Such collaborations lead to groundbreaking discoveries and innovations in CADD [155]. While collaborations offer numerous benefits, they are not without challenges. Issues related to data privacy, intellectual property rights, and varying regulatory standards can be impediments. Addressing these challenges requires meticulous planning and robust legal frameworks [156]. While the path to effective drug discovery is arduous, collaborative endeavors promise to make the journey shorter and more fruitful. Through united efforts, the most formidable challenges in CADD will be surmounted.

## 14. CADD in Personalized Medicine: Tailoring Therapies to Individuals

Personalized medicine, often interchangeable with precision medicine, seeks to customize healthcare by tailoring decisions and practices to the individual patient. Integrating CADD with personalized medicine stands to revolutionize treatment paradigms [157]. The completion of the Human Genome Project has provided a detailed genetic blueprint. Leveraging this information, CADD can help design drugs targeting specific genetic mutations or variants associated with diseases [158]. By integrating genetic, epigenetic, and proteomic data, CADD tools can forecast a patient’s likely response to a drug. This facilitates the administration of therapies that are most likely efficacious while minimizing adverse effects [159]. In some rare diseases caused by particular genetic mutations, CADD offers the possibility of creating drugs tailored for individual patients, an approach that would be the pinnacle of personalized medicine. Biomarkers are vital in personalized medicine, providing measurable indicators of disease states. CADD aids in the discovery of drugs that can modulate these biomarkers, leading to personalized therapeutic solutions. As electronic health records become more prevalent, integrating this real-world data with CADD models can provide insights into drug performance in diverse populations, allowing for more individualized therapy recommendations.

The prospects of personalized medicine via CADD are exciting, but they come with ethical dilemmas, especially regarding data privacy and potential inequalities in access to tailored treatments. In sum, CADDs intersection with personalized medicine promises treatments optimized for each patient, transcending the one-size-fits-all approach. A new era of healthcare beckons by harnessing the power of computational tools in sync with individual data. 

Often called theranostics, this approach leverages CADD to develop drugs alongside diagnostic tests that determine a patient’s suitability for the treatment. This ensures the right drug reaches the right patient at the right time. Personalized medicine is greatly enhanced by patient-derived models like organoids or patient-derived xenografts. CADD can use data from these models to simulate drug responses, allowing individualized therapy adjustments [160]. Cancer epitomizes the need for personalized medicine, given the heterogeneity in tumors, even within the same cancer type. CADD tools can analyze tumor genomic data to identify druggable targets unique to each patient’s cancer profile [161]. As wearable technology becomes increasingly sophisticated, capturing diverse health metrics and integrating this data with CADD models can fine-tune drug recommendations based on real-time patient status [162]. While the prospects of CADD-driven personalized medicine are revolutionary, the associated costs are a concern. Ensuring these tailored treatments are economically viable and accessible to all, regardless of socio-economic status, is a pressing challenge [163]. Integrating CADD with personalized medicine could redefine treatment regimens, ensuring patients receive interventions tailored to their unique genetic and physiological profiles. But as with all transformative advances, balancing innovation with ethics, accessibility, and cost remains pivotal.

## 15. Elevating Drug Design: The Convergence of AI, Machine Learning, and CADD

Artificial intelligence (AI) and machine learning (ML) have recently made substantial inroads into multiple scientific disciplines. Their intersection with Computer-Aided Drug Design (CADD) is yielding transformative changes in drug discovery processes [164].

A subset of machine learning, deep learning, especially with convolutional neural networks (CNNs), has demonstrated proficiency in predicting drug properties, analyzing molecular structures, and optimizing molecular design [165]. AI-driven models can predict drug-drug interactions, offering insights into potential synergies or adverse reactions when multiple drugs are co-administered [134]. By examining vast databases of drug properties and clinical outcomes, AI models have been instrumental in identifying new therapeutic applications for existing drugs [166]. Quantitative structure-activity relationship (QSAR) models benefit from ML by enabling more accurate predictions of a molecule’s biological activity based on its chemical structure [167]. High-throughput screening of vast molecular libraries can be expedited using AI, narrowing down potential drug candidates in a fraction of the time traditional methods require [168]. AI can assist in the design of novel drug molecules from scratch, tailoring them to have desired properties while minimizing potential side effects [169]. While AI and ML offer exciting prospects in CADD, they are not devoid of challenges. Data quality, overfitting, interpretability, and the need for extensive computational resources are areas of concern [66]. In conclusion, AI and ML are reshaping the landscape of drug discovery. By combining the computational prowess of these technologies with the methodological rigor of CADD, the promise of more effective, safer, and tailor-made drugs seems closer than ever before.

## 16. Conclusions

In conclusion, Computer-Aided Drug Design is a transformative catalyst in modern drug discovery, poised at the intersection of biological intricacies and computational prowess. The journey from historical breakthroughs to the contemporary landscape underscores its pivotal role in expediting drug development. However, as CADD charts its future trajectory, challenges emerge, necessitating continual optimization, ethical considerations, and the integration of diverse biological data. Success stories exemplify the tangible impact of CADD on clinical applications, while the infusion of Machine Learning augments predictive capabilities, unveiling new frontiers. Collaborative networks and global initiatives democratize drug discovery, emphasizing the strength of unity. The convergence of personalized medicine offers tailored solutions, albeit with ethical and accessibility challenges. Looking ahead, quantum computing, immersive technologies, and green chemistry promise a paradigm shift demanding a delicate balance between innovation and ethical responsibility. Collaborative platforms and open-source initiatives serve as beacons of hope, emphasizing shared knowledge in a global context. Ethical and regulatory considerations are pivotal in guiding CADDs responsible evolution, especially as it converges with emerging technologies and navigates the complexities of the digital era. The symbiosis of experimental data and CADD enriches drug discovery, highlighting the synergistic relationship between computational predictions and real-world validations. In education, the integration of CADD training becomes essential for shaping proficient individuals capable of navigating the multidisciplinary landscape of drug discovery. As CADD anticipates accuracy, bias mitigation, and sustainability challenges, proactive measures must be taken to ensure responsible and compliant use. In essence, the trajectory of CADD is a journey of innovation, challenges, and ethical considerations, paving the way for a future where drug discovery is faster, more precise, and more patient-centric, ultimately contributing to a healthier tomorrow.

## Figures and Tables

**Table 1:** Structure-predicting methods and tools.

Methods	Programs
Homology Modeling/Comparative Modeling: Create a 3D model of the target protein using a homologous protein’s empirically confirmed structure as a guide.	MODELLER, SWISS-MODEL, Phyre2, RaptorX, I-TASSER
Ab Initio Modeling: Build a 3D model of the target protein by sampling the protein’s conformational space without using experimental data.	Rosetta, QUARK, AlphaFold, ESMFold, PCONS5
Threading: Build a 3D model of the target protein by aligning the protein sequence with the sequences of proteins of known structure.	MUSTER, 3D-PSSM, LOMETS, HHpred
Hybrid Modeling: Combine two or more modeling approaches to improve the accuracy of the predicted structure.	CABS-flex, PrimeX, GalaxyHomomer
Molecular Dynamics: Simulate the behavior of the protein over time using classical or quantum mechanics.	GROMACS, NAMD, CHARMM
Knowledge-based methods: Use existing knowledge about protein structure and function to predict the structure of the target protein.	ProSMoS, ProQ3D, I-TASSER-2GO
Template-free methods: Build a 3D model of the target protein without using templates or homologous proteins.	CONFOLD2, MetaPSICOV, TrRosetta
Fragment-assembly methods: Build a 3D model of the target protein by assembling fragments of known protein structures.	PEP-FOLD3, Robetta, QUARK

**Table 2:** Docking Tools, their advantages, and disadvantages.

Tool	Application	Advantages	Disadvantages
AutoDock Vina	Predicting the binding affinities and orientations of ligands.	Fast, accurate, and easy to use.	May not be as accurate for complex systems.
AutoDock GOLD	Predicting the binding affinities and orientations of ligands, especially for flexible ligands.	Accurate for flexible ligands.	Requires a license and can be expensive.
Glide	Predicting the binding affinities and orientations of ligands.	Accurate and integrated with other Schrödinger tools.	Requires the Schrödinger suite, which can be expensive.
DOCK	Predicting the binding affinities and orientations of ligands and performing virtual screening.	It is versatile and can be used for both docking and virtual screening.	Can be slower than other tools.
LigandFit	Predicting the binding affinities and orientations of ligands.	Easy to use and integrated with other Schrödinger tools.	May not be as accurate for complex systems.
SwissDock	Predicting the binding affinities and orientations of ligands.	Easy to use and accessible online.	May not be as accurate for complex systems.

## Data Availability

Data sharing is not applicable.
